# Modulation of Habit Formation by Levodopa in Parkinson's Disease

**DOI:** 10.1371/journal.pone.0027695

**Published:** 2011-11-16

**Authors:** Frank Marzinzik, Johann Wotka, Michael Wahl, Lea K. Krugel, Catarina Kordsachia, Fabian Klostermann

**Affiliations:** 1 Department of Neurology, Campus Benjamin Franklin, Charité-University Medicine Berlin, Berlin, Germany; 2 Institute of Psychology, Humboldt-University Berlin, Berlin, Germany; Charité-Universitätsmedizin Berlin, Germany

## Abstract

Dopamine promotes the execution of positively reinforced actions, but its role for the formation of behaviour when feedback is unavailable remains open. To study this issue, the performance of treated/untreated patients with Parkinson's disease and controls was analysed in an implicit learning task, hypothesising dopamine-dependent adherence to hidden task rules. Sixteen patients on/off levodopa and fourteen healthy subjects engaged in a Go/NoGo paradigm comprising four equiprobable stimuli. One of the stimuli was defined as target which was first consistently preceded by one of the three non-target stimuli (conditioning), whereas this coupling was dissolved thereafter (deconditioning). Two task versions were presented: in a ‘Go version’, only the target cue required the execution of a button press, whereas non-target stimuli were not instructive of a response; in a ‘NoGo version’, only the target cue demanded the inhibition of the button press which was demanded upon any non-target stimulus. Levodopa influenced in which task version errors grew from conditioning to deconditioning: in unmedicated patients just as controls errors only rose in the NoGo version with an increase of incorrect responses to target cues. Contrarily, in medicated patients errors went up only in the Go version with an increase of response omissions to target cues. The error increases during deconditioning can be understood as a perpetuation of reaction tendencies acquired during conditioning. The levodopa-mediated modulation of this carry-over effect suggests that dopamine supports habit conditioning under the task demand of response execution, but dampens it when inhibition is required. However, other than in reinforcement learning, supporting dopaminergic actions referred to the most frequent, i. e., non-target behaviour. Since this is passive whenever selective actions are executed against an inactive background, dopaminergic treatment could in according scenarios contribute to passive behaviour in patients with Parkinson's disease.

## Introduction

Replacement of depleted dopamine (DA) is the central principle in the treatment of Parkinson's disease (PD) [1], [2] with beneficial motor, but less predictable non-motor effects. This discrepancy can be explained by the preponderance of PD-related DA deficiency in specific brain regions [3]. Over a long period after disease onset, nigral projections to dorsal parts of the striatum are worst affected leading to the major motor symptoms [4], while other dopaminergic networks remain comparably intact [5]. Accordingly, DA replacement for the movement disorder may overdose brain areas without relevant DA deficit [6]–[9].

In this context, DA-dependent functions of the mesocortical system, spanning from the ventral tegmental area to frontal regions, are of particular interest. This network is strongly involved in learning processes [10]–[12] and, in PD patients, has been related to the development of impulsive-compulsive disorders, understood as the consequence of enhanced learning from positively reinforced actions under dopaminergic drugs [13]–[18].

Little is known about the influence of DA replacement on non-reinforced learning, but hypotheses may be derived from some general functions attributed to DA in corticobasal networks. DA has been proposed to regulate the trade off between stability and flexibility. Specifically, high striatal levels are thought to support the flexible adaptation to changing environmental conditions, whereas high mesocortical concentrations seem to stabilise ongoing behavioural goals [19]–[21]. With respect to learning patterns in PD, this could mean that in treated PD patients excessively driven mesocortical functions would fixate acquired behaviour, whereas striatal DA depletion in untreated patients should slow down gradual learning from changing environmental rules.

To test these assumptions, the performance of PD patients on versus off levodopa and of healthy controls was analysed in a task, requiring reactions to seldom target cues intermingled between frequent non-target stimuli without feedback control. The target cues were first preceded by one out of several non-target stimuli (conditioning phase) and then presented in random order (deconditioning phase), resembling the general structure of Nissen & Bullemer's serial reaction time (SRT) task for the assessment of implicit learning [22]. This SRT aspect was embedded in a Go/NoGo design, since in reinforcement learning high DA levels support the learning of rewarded actions, but unfold detrimental effects when inhibition learning is required [9], [13], [16], [17], [23], [24]. In order not to miss a similar DA-dependent modulation in non-reinforced learning, the paradigm was presented with two instructions: first, in a ‘Go version’, target cues were instructive for the release of a button press, whereas non-target stimuli did not require any response, and, second, in a ‘NoGo version’ only upon target cues the button press had to be withheld, while it was required upon all non-target stimuli.

Under the basic idea that performance declines during deconditioning could indicate the carry-over of (no longer valid) rule representations built up during conditioning, reaction times and error rates were compared between these task phases.

## Methods

### Participants

Sixteen patients participated in the current study (11 male, 5 female; 64.4±6.6 years of age; 14.0±3.7 years of education [mean ± standard deviation]). They were recruited from the Outpatient Clinic for Neurological Movement Disorders of the Charité (Campus Benjamin Franklin) and fulfilled the Brain Bank Criteria for PD. From the patients sent to the Outpatient Clinic, those with levodopa monotherapy (772±618 mg) were selected to avoid confounding effects from other antiparkinson drugs, and experiments were conducted before further drug adjustments. Exclusion criteria were the presence of neurological disease apart from PD, the intake of drugs with central mechanisms of action besides levodopa, and less than 24 points in the Mini Mental State Examination (MMSE) [25]. Further, 14 healthy subjects (8 male, 6 female; 68.4±4.5 years of age; 15.6±2.8 years of education), free from the aforementioned exclusion criteria, took part in the experiment as controls.

All participants were right-handed, as assessed by Oldfield's handedness inventory [26]. Further, the scores for the motor part of the Unified Parkinson's Disease Rating Scale (UPDRS, part 3), the Beck Depression Inventory (BDI) and the Fatigue Severity Scale (FSS) were determined to delineate putative cognitive, affective and fatigue-related disorders [27]–[29]. The data are summarized in Table 1. All participants gave written informed consent to the study protocol approved by the Ethics Committee of the Charité. Since we aimed at comparing effects of levodopa medication, patients engaged in the experiments twice, once under treatment (on condition) and once after overnight withdrawal (off condition) with a minimum interval of 4 weeks between both sessions. The medication state, in which patients first accomplished the experiment, was balanced for both task conditions.

**Table 1:** Clinical data.

	UPDRS	BDI	MMSE	FSS
**Controls**	0.9±1.2	5.2±3.2	29.1±0.9	28.4±12.5
**PD-on**	19.5±−7.8	6.2±5.6	28.1±1.8	28.6±15.5
**PD-off**	29.5±−9.7	6.9±3.7	28.4±1.9	28.5±14.3

Patients differed between on and off levodopa states and from controls with respect to the scores in the Unified Parkinson's Disease Rating Scale (UPDRS). Normal values without significant differences between groups were obtained in the Beck Depression Inventory (BDI), the Mini Mental State Examination (MMSE) and the Fatigue Severity Scale (FSS). All data are provided as mean values ± standard deviation.

### Experimental procedure

The participants engaged in two task conditions, demanding selective reactions to visually presented target and non-target signals. In the Go version of the task, they had to respond to a predefined target signal by a right-finger button press, whereas responses should not be given to any of the other signals (non-targets). In contrast to this, in the NoGo version the right-finger button press had to be performed upon any non-target signal, whereas only upon the target signal this response had to be withheld.

**Figure 1:**
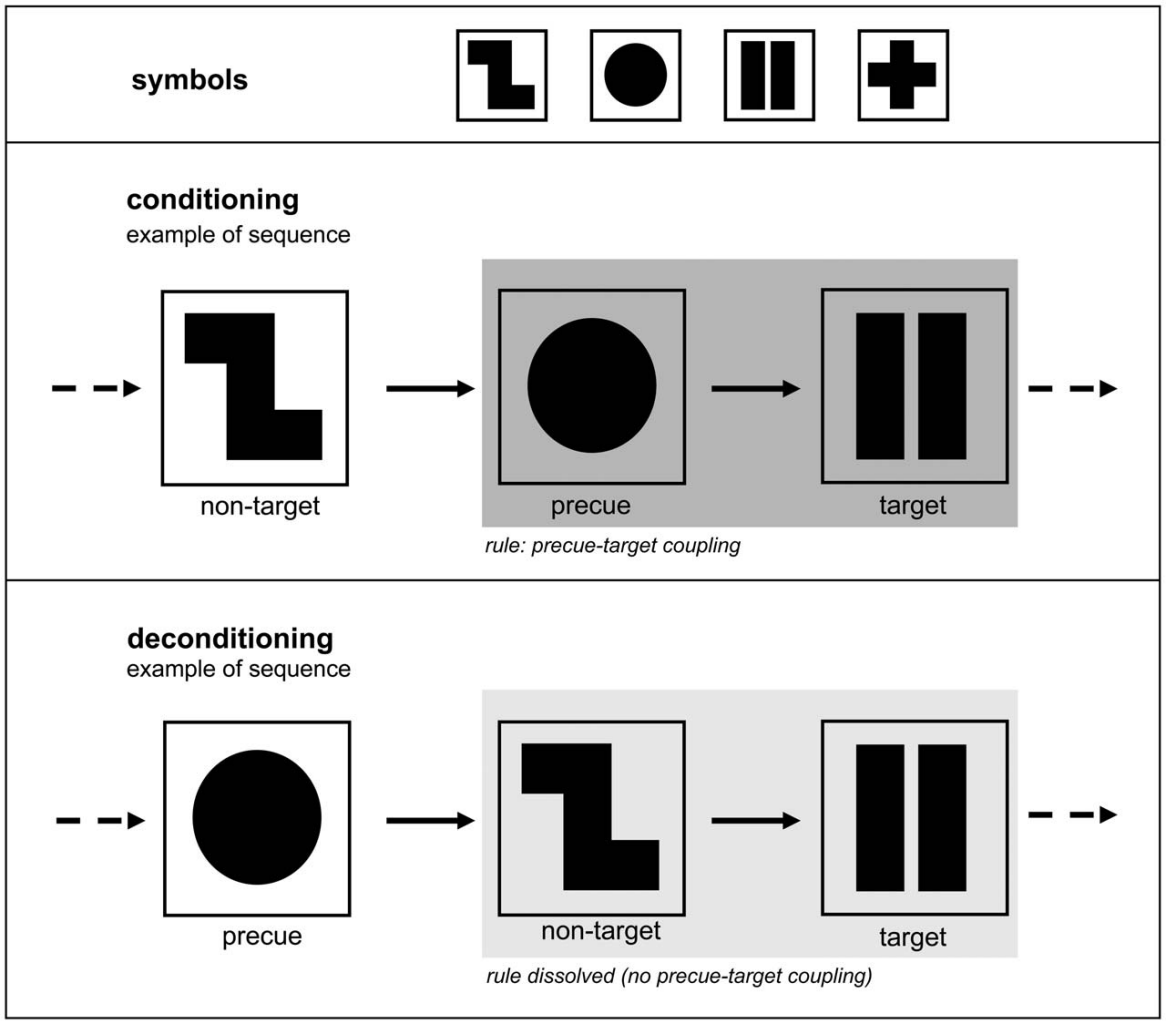
Task structure. Four neutral and equiprobable symbols were presented in pseudorandomised order, one of which was defined as target signal. During a conditioning phase of 120 signal presentations, the target was always preceded by one of the three non-target signals (precue). Over the subsequent 40 presentations, this precue-target coupling was dissolved (deconditioning phase). The conditioning-deconditioning sequence (comprising 160 presentations) was repeated five times with alternating precues. To avoid conscious recognition of the task structure, one-minute pauses were held every 200 trials. Thus, conditioning and deconditioning phases never appeared at the same point in time with respect to the breaks.

In both the Go and NoGo task version four equiprobable visual stimuli occurred, one of which was the target and three of which were non-targets. Over blocks of 120 signals (conditioning), the target signal was consistently preceded by the same non-target signal (in the following labelled precue). After each conditioning block, this coupling was dissolved, i. e., the former precue did no longer precede the target cue for a block of 40 signals (deconditioning). Every 160 signals (conditioning plus deconditioning) a new conditioning phase with another precue (but constant target) began. Five alternating conditioning/deconditioning phases were run, overall containing 800 presentations (cf. Fig. 1).

Importantly, participants should not become aware of the alternating structure of the task. To this end, every 200 trials, pauses of one minute were held. In so doing, conditioning and deconditioning phases never appeared at the same point in time with respect to the breaks to avoid conscious perception of the task rules and trend effects from decaying vigilance or attention.

Subjects sat at 1.5 m from a 17′ computer screen with the index finger comfortably positioned over a push-button on the right-hand armrest. All stimuli popped up within a constantly present 6×6 cm frame in the middle of the screen. The presentation time of target and non-target signals (including precues) was set to 175 ms, the interstimulus interval to 1000 ms.

### Statistical Analysis

Demographic data and clinical score values were compared between control subjects and patients (on versus off levodopa) with unpaired and, paired *t*-tests, respectively. For the analysis of response latencies (accepted within a range of 150 to 900 ms after Go stimuli) and accuracy of task performance, two-way ANOVAs were run with respect to reaction times and error rates if the data met criteria for parametric testing due to Kolmogorov-Smirnow and Levene testing. *Group* was included as three-level test factor (control subjects/patients off levodopa/patients on levodopa) and *Learning Phase* as a test factor with four levels, specified as equally long segments of performance throughout each block of conditioning and subsequent deconditioning (performance over stimulus 1–40/41–80/81–120/121–160, the latter segment being the deconditioning phase). In case of sphericity violations, Huynh-Feldt corrections were performed. Post-hoc comparisons were run as t-tests.

The rationale behind treating results from the same PD patients on versus off levodopa as stemming from different groups was that we aimed at the broadest possible analysis of medication-dependent modulation of normal task performance, together with a comprehensive assessment of putative interactions of the test factors. Importantly, this statistical approach is particularly conservative, since it minimises the risk of erroneously assuming differences between treated and untreated PD patients. The reason for this is that the statistical assumption of data variance is larger for cohorts with distinct than with identical subject, so that the treatment of within-subject as between-subject information overestimates data analogousness.

Only in the deconditioning phase of the Go version, errors to target cues in patients on levodopa were not normally distributed. Accordingly, we abstained from running an ANOVA in this case, and compared target responses during conditioning and deconditioning with the non-parametric Wilcoxon-test.

## Results

With respect to demographic and non-motor scores (age, years of education, BDI, FSS, MMSE) no differences were obtained between patients and control subjects or between patients on and off levodopa (*p*>.05). Only in regard to the motor UPDRS, we revealed an expected statistical distinction both between patients on and off levodopa as well as between patients in both medication states and controls (*p*<.01).

In the debriefing procedure held as a standardized oral interrogation after the experiment none of the participants could report on any coupling of signals or on the alternation of task sequences, which suggests that the task structure remained hidden.

### Reaction Time (RT)

**Figure 2:**
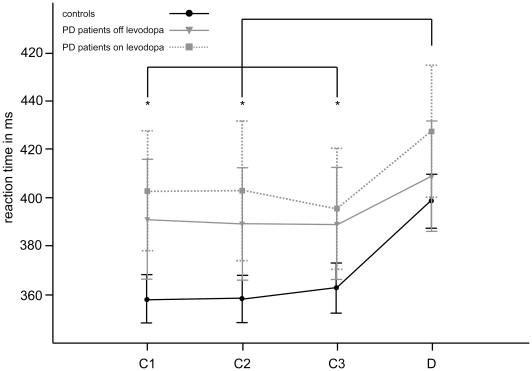
Reaction times during conditioning and deconditioning. Average reaction times are displayed per group over blocks of 40 presentations, exemplified by the responses to non-target cues in the NoGo task version with the highest number of responses (no difference was obtained between task versions). Since conditioning-deconditioning sequences comprised 120 presentations during conditioning followed by 40 presentations during deconditioning, block 1 to 3 (labelled C1, C2 and C3) reflect performance during conditioning, whereas block 4 (labelled D) equates to deconditioning. The error bars indicate the standard error of the mean. Note that reaction times increased significantly during deconditioning compared to any of the conditioning blocks (indicated by asterisks) over all groups.

In both the Go and NoGo condition of the task, ANOVAs proved *Learning Phase* to be a main factor for RT [Go: *F*(1.9, 73) = 12.36, *p*<.01; Nogo: *F*(3, 129) = 20.38, *p*<.01]. Post-hoc tests demonstrated that this effect, concerning target reactions in the Go condition and non-target reactions in the NoGo condition, was due to significant increases of response latencies in the last segment of each block, i. e. during deconditioning as compared to the conditioning phase (deconditioning versus conditioning block 1 to 3 for Go: *p*<.05; for NoGo: *p*<.01). No significant differences were obtained across the different conditioning blocks. Although on average controls responded somewhat faster than PD patients on and off levodopa, *Group* was not identified as a factor of RT. Further, no interaction between *Learning Phase* and *Group* was found (cf. Fig. 2). No response had to be excluded based on the criterion of response latencies between 150 to 900 ms after Go stimuli.

### Accuracy

**Figure 3:**
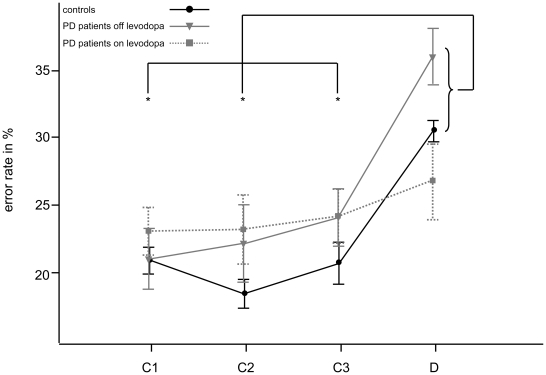
Errors in the NoGo version of the task. In the NoGo version of the task, erroneous reactions to target signals were executed responses (commission errors). The average rate of target commission errors is displayed per group over blocks of 40 presentations over the 120 presentations during conditioning (blocks 1 to 3, labelled C1, C2 and C3) and the subsequent 40 presentations during deconditioning (block 4, labelled D). The error bars indicate the standard error of the mean. Note the significant increase of commission errors during deconditioning compared to any of the conditioning blocks in healthy subjects and patients with Parkinson's disease off levodopa (indicated by asterisks), whereas in patients on levodopa this effect could not be detected.

In the NoGo version of the task, the ANOVA of errors to target stimuli revealed *Learning Phase* to be a main factor [*F*(2, 86.2) = 20.5, *p*<.01]. Further, *Group* interacted with *Learning Phase*, demonstrating that errors did not occur uniformly across groups [*Group*×*Learning Phase*: *F*(4, 86.2) = 2.5, *p*<.05]. Post-hoc tests demonstrated that the interaction *Group*×*Learning Phase* was due to the fact that in control subjects and patients off levodopa erroneous target reactions increased during deconditioning, whereas this was not the case in PD patients on levodopa. Thus, in the NoGo version of the task, only unmedicated patients and healthy persons tended to respond to target cues during deconditioning when the suppression of this reaction was actually demanded (deconditioning versus learning block 1 to 3 for PD patients off levodopa/control subjects: *p*<.01/.05; same comparisons for PD patients on levodopa: *p*>.05; cf. Fig. 3).

**Figure 4:**
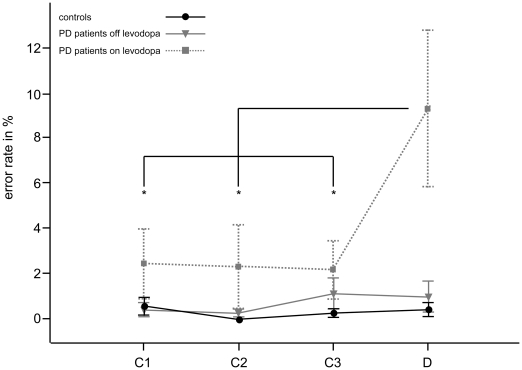
Errors in the Go version of the task. In the Go version of the task, erroneous reactions to target signals were response omissions. The average target omission rate is displayed per group over blocks of 40 presentations over the 120 presentations during conditioning (blocks 1 to 3, labelled C1, C2 and C3) and the subsequent 40 presentations during deconditioning (block 4, labelled D). The error bars indicate the standard error of the mean. Note the significant increase of response omissions during deconditioning compared to any of the conditioning blocks in patients with Parkinson's disease on levodopa (indicated by asterisks), which was not found in patients in and patients off levodopa and healthy subjects.

In the Go version of the task, errors to target stimuli were not normally distributed in patients on levodopa in the deconditioning phase and, accordingly, an ANOVA was not run in this case. Instead, comparisons equivalent to those for the NoGo version of the task were performed. In controls and patients off levodopa t-tests did not show any differences between errors, neither between the different conditioning blocks nor between conditioning blocks and deconditioning. In patients on levodopa, however, errors increased during deconditioning compared to all conditioning blocks (Wilcoxon-testing of deconditioning vs. conditioning blocks 1 to 3: *p*<.05). Between the conditioning blocks no change of erroneous target responses was identified. Thus, only PD patients on levodopa tended to omit required target responses in the deconditioning phase of the Go version of the task. This is summarised in Fig. 4.

No effects of conditioning or deconditioning were identified by the ANOVAs for non-target reactions, neither in the Go nor in the NoGo version of the task. However, it was assumed that the overall analysis of all non-target reactions could have blurred the statistical proof of main factors, given that only specific stimulus sequences were expected to induce erroneous non-target reactions. Therefore, it was additionally analysed if during deconditioning the error rate after stimuli following former precues was higher than the error rates to non-target stimuli which were not preceded by former precues. This would have indicated that behaviour relying on the main conditioning rule, namely to provide a target reaction to stimuli after precues, had been carried over to the deconditioning phase (in which former precues preceded non-target stimuli, thus potentially facilitating erroneous target reactions). Worthwhile noticing, however, respective t-tests did not reveal any such effect.

Altogether, in the analysis of performance accuracy a dissociation of erroneous target reactions was found between Go and NoGo condition with healthy subjects and patients off levodopa on the one hand and patients on levodopa on the other hand.

## Discussion

This study aimed at analysing effects of central DA supply on non-reinforced learning. Therefore, PD patients on and off levodopa as well as healthy subjects engaged in a Go/NoGo task with alternating conditioning and deconditioning of target cues. According to assumptions on DA in corticobasal systems, it was hypothesised that unmedicated PD patients would be impaired in learning from conditioning, reflecting striatal DA deficiency with slowed acquisition of environmental rules [20]. Contrarily, we predicted that patients on levodopa would overperpetuate conditioned actions, reflecting excessive stabilisation of behavioural patterns under unphysiological dopaminergic drive of mesocortical regions [19], [21]. In order not to miss interactions of DA supply and task instructions, as have been described for reinforcement learning [9], [13], [16], [17], [23], [24], these assumptions were tested under the opposite demands to selectively execute (Go version of the task) and to inhibit responses (NoGo version of the task).

The following main findings were obtained: (i) concerning reaction time (RT), a uniform increase of response latencies was observed during deconditioning compared to conditioning; this effect was not dependent on DA replacement in PD, the disease itself or the task instruction; (ii) on the level of accuracy, task performance decreased from conditioning to deconditioning, but this effect was influenced by the medication state of PD patients and dependent on the task instruction; (iii), task performance in patients on levodopa was principally different from that of patients off levodopa and healthy subjects, the latter groups behaving similarly.

The expectation that the cancellation of target precueing would slow down response latencies was confirmed, whereas the assumption that conditioning itself would - in a process of gradual learning during rule repetition - decrease RT was not fulfilled, since no change occurred over the sequential conditioning blocks in whatever task version. This absence of direct conditioning effects may be explained by the plainness of demanded responses: RT for simple button presses may have been saturated ab initio so that predictive task elements could not further accelerate response latencies, whereas during deconditioning the violation of the conditioned expectations led to RT deceleration. The assumption of primarily saturated response speed appears also compatible with the observation that RT in PD patients, who disproportionally slow down under more intricate response demands, did not significantly diverge from RT in healthy subjects [30]–[32].

The RT results did not support the hypothesis of a DA role in non-reinforced learning, since deceleration in deconditioning occurred uniformly across study participants, i. e., subjects were affected by the withdrawal of target precueing independently from their group affiliation.

Concerning performance accuracy, however, specific interactions of levodopa treatment and the task instruction were found. Principally, error rates differed between conditioning and deconditioning phases and not between sequential conditioning blocks, but unlike for the RT results, these differences depended on whether PD patients were under medication with levodopa or not. Whereas in patients on levodopa errors increased during deconditioning in the Go version of the task (as omissions of required target responses), this was not the case in patients off levodopa and healthy subjects. Contrarily, in the latter groups errors increased in the NoGo version of the task (as commission errors to target stimuli which required response suppression), while this effect was absent in patients on levodopa.

For interpreting these results, it should also be noted that error increases during deconditioning were only found after the presentation of target cues, but not upon non-target stimuli. Increases of erroneous reactions to non-target stimuli, however, could have been expected, since precues in the conditioning phases of the task were the only stimulus class with full predictivity for the subsequent occurrence of target stimuli. Accordingly, during deconditioning ‘target responses’ to non-target stimuli (after former precues) would have indicated maintained adherence to the expired coupling rule. That this was not the case, but that levodopa instead changed reaction tendencies to target stimuli raises the question on which processes the treatment eventually acted.

In this regard, it is interesting that in implicit, non-reinforced learning, as studied here, environmental rules are extracted from repetitive information related to prevailing behaviours [33]. Against this background, the expected representation of precue-target coupling seems only one possible strategy for the formation of behaviour. Alternatively, the rules related to stimuli which are predictive of the habitual, i. e., non-target behaviour might be targeted by a more global learning approach. In this formulation, task participants would first of all learn the predictivity of all non-precues, being seventy five percent of all stimuli, for subsequent non-target reactions. This would define the increased tendency to provide ‘non-target reactions’ to target stimuli in deconditioning, being the only task phase in which non-target stimuli could be followed by target cues. However, while the strengthening of this process by PD treatment can be understood within the concept of DA-dependent stabilisation of behaviour [19], [21], it remains to be settled why levodopa intake should reverse respective effects, which in controls and in untreated patients have been observed only in the NoGo task version.

Worthwhile noticing, the overall error rate was several-fold higher in the NoGo than in the Go version of the task. This suggests a relation between the particular error increase during deconditioning and task difficulty in controls and untreated PD patients. A simple explanation for this could be that perpetuated response tendencies, leading to erroneous reactions, cannot be adequately controlled, if high cognitive effort has to be spent on proper task accomplishment (as in NoGo), whereas the resources for such control can be mobilised in cognitively less demanding tasks (as in Go). Concerning the levodopa-mediated reversal of this effect, interactions of DA supply and task instructions seem to come into play: high DA levels unfold a negative impact on reinforced inhibition learning [9], [13], [16], [17], [23], [24] and, accordingly, the lack of carry-over errors in the NoGo task version in treated PD patients could indicate attenuated learning when the demand was to selectively suppress habitual responses. Contrarily, when the selective execution of a response was required, high DA levels in medicated PD patients could have mediated overshooting stabilisation of the prevalent behaviour, explaining the occurrence of carry-over errors even in the relatively simple Go version of the task.

We did not find PD itself to impair habit conditioning as a consequence of striatal DA depletion [20]. Thus, the overall results support the idea of disequilibrating still intact DA-dependent functions by pharmacological replacement therapy in PD [6]–[9]. Candidate target regions for the present overdose effects are ventral fronto-striatal and mesocortical dopaminergic networks — a challenging issue for additional neuroimaging studies.

In conclusion, DA states influenced task performance depending on the given instruction, but contrary to reinforcement learning [13]–[15], high DA levels appear to enhance the most frequent reaction type rather than selectively demanded responses, when action feedback is unavailable. This is also of interest from a clinical perspective, because dopaminergic replacement therapy in PD is usually associated with the evolution of impulsive-compulsive behaviour [34]–[43]. Nevertheless, the present findings suggest that, if neutral actions are carried out against a background of inactivity, replacement therapy might also foster passive behavioural tendencies. As these are commonly considered disease-inherent rather than drug-induced in PD [44]–[46], clinicians should be sensitised not only to what patients under dopaminergic treatment excessively do, but also to what they *not* do.
